# Effect of Chemical Grafting Parameters on the Manufacture of Functionalized PVOH Films Having Controlled Water Solubility

**DOI:** 10.3389/fchem.2017.00038

**Published:** 2017-06-19

**Authors:** Andreas Haas, Daniel Schlemmer, Uwe Grupa, Markus Schmid

**Affiliations:** ^1^Materials Development, Fraunhofer Institute for Process Engineering and Packaging IVVFreising, Germany; ^2^Department of Food Technology, Hochschule FuldaFulda, Germany; ^3^TUM School of Life Sciences Weihenstephan, Technical University of MunichFreising, Germany

**Keywords:** poly(vinyl alcohol), solubility, biodegradable packaging, extrusion, chemical grafting, fatty acid chloride, hydrophobic surfaces

## Abstract

This study investigated the chemical grafting of a single-layer poly(vinyl alcohol) (PVOH) film. The effect of the grafting parameters (grafting time, grafting temperature, and concentration of fatty acid chloride) on the hydrophobicity of the film surface and the film solubility were evaluated. The PVOH substrate film (cold-water soluble at 20°C) was manufactured by flat extrusion and had a thickness of 50 μm (±5 μm). The chemical grafting was performed using the transfer method with palmitoyl chloride (C_16_). The solubility, surface energy, and water vapor transmission rate of the grafted films were measured. The process parameters which produced the most hydrophobic PVOH film were found to be a fatty acid concentration of 3%, a grafting time of 14 min, and a grafting temperature of 130°C. These studies involved systematic adjustment of the hydrophobicity of one side of PVOH films. The results open up opportunities for packing fluids in water soluble packaging.

## Introduction

Water-soluble packaging is used for a wide range of products, including detergents and laundry bags in hospitals (Pardos, 2004; Elsner and Domininghaus, 2008). This type of packaging enables the secure handling of a product, greatly reducing health hazards by avoiding contact with harmful products (Farmer, 2013). In addition, chemicals packed in a water-soluble film are easy to dispense. At present, the maximum water content of fluids packed in a water-soluble film is 5–7% (Dickler and Ruck, 2000). The present study aimed to develop a water-soluble single-layer film for packing fluids having water contents greater than 5% (w/w) which fully dissolve when contacted with water (Pardos, 2004; Morris, 2017). The substrate material used for the packaging film was plasticized poly(vinyl alcohol) (Solovyov and Goldman, 2008) which was cold water soluble, biodegradable, and compostable (Nayak, 1999).

**Graphical Abstract 1:**
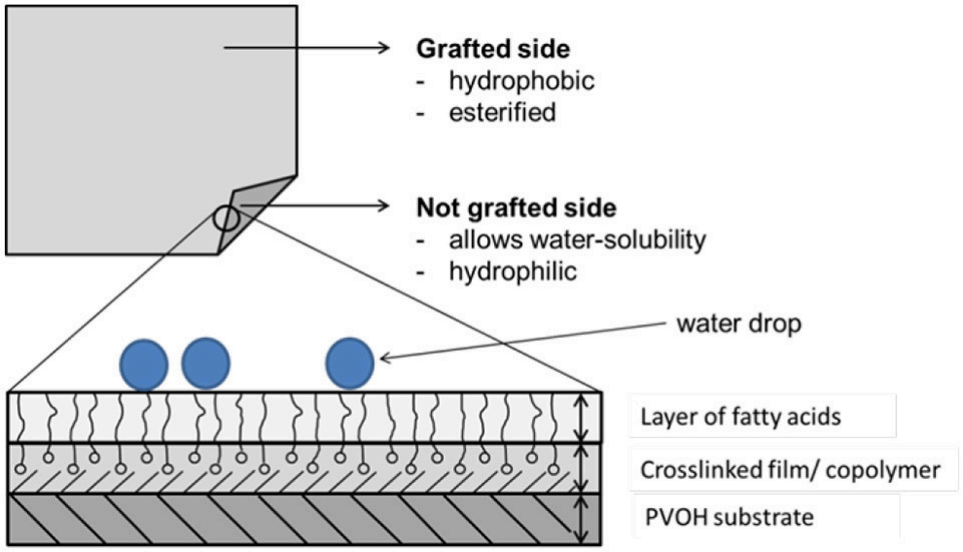
Polymer film with the grafted layer on the upper film side.

Fatty acid chlorides have the highest reactivity of the fatty acid halides. This is the reason they were used for the grafting process (Gunstone, 2007). They are liquid at room temperature and have chain lengths of between 6 and 24 monomers. Fatty acid chlorides can be either saturated or unsaturated. The molecules have a polar carboxyl group at one end and a non-polar carbonyl group at the other end, enabling grafting to produce a hydrophobic effect (Ullmann, 2005; Schormüller, 2014). In general, poly(vinyl alcohol) is used as a sizing agent in the paper industry, for coating yarns in the textile industry, and also for adhesive preparation. Another large-scale use of PVOH is in the cosmetics industry as an excipient for shampoos, salves, and dense media. It is also used as a coating material and as a partial layer in multilayered polymer film composites. Water-soluble PVOH films are used for packaging laundry detergents, dyes, agricultural chemicals, and cleaning agents. The advantage of water-soluble packaging is the delivery of pre-dosed quantities of chemicals and safe handling of harmful substances (Misra, 1993; Olabisi, 1997; Mishra et al., 2009; Peters, 2011).

Industrially manufactured PVOH polymers are produced from polyvinyl acetates by alkali-catalyzed transesterification in solution (Ebnesajjad, 2013a). Poly(vinyl alcohol)s are typically characterized by the degree of polymerization of the starting materials (molecular weight), the degree of hydrolysis, and the saponification number (Kruse, 1997). They belong to the group of polymers which undergo biodegradation, but these properties depend on the type and composition (Andrady, 2003; Pająk et al., 2010; Kasirajan and Ngouajio, 2012; Negim et al., 2014). Vinyl alcohol monomers do not exist in a stable form in nature, so they have to be synthesized in a two-step process. The first step is the polymerization of vinyl acetate monomers to form polyvinyl acetate. The second step is the hydrolysis of the polyvinyl acetate to poly(vinyl alcohol) (Tímár-Balázsy and Eastop, 1998; Hassan and Peppas, 2000; Beswick and Dunn, 2002).

The general formula of fatty chlorides is R'COX, where X represents the halogen. Experiments using different fatty acid chlorides were carried out by Samain (2002) and Schmid et al. (2012, 2014). Palmitoyl chloride (C_16_) and stearoyl chloride (C_18_) turned out to be particularly suitable fatty acid chlorides for the chemical grafting process and the ability to make materials hydrophobic (Samain, 2002). Blended films based on PVOH are low-cost and biodegradable. PVOH is compatible with other polymers because of the presence of OH-groups, namely sites for bonding. PVOH films are mostly manufactured by extrusion processes. Plasticizers, fillers, and pigments are often added to the PVOH because of the difficulty processing pure PVOH. The PVOH film used in the present study had an average thickness of 50 μm and was modified by a chemical grafting process using a fatty acid chloride in order to give the film hydrophobic characteristics. The solubility, surface energy, and water vapor transmission rate of the modified films were measured (Olabisi, 1997).

The aim of the study was to investigate how and to what extent the grafting process parameters, namely the temperature, time, and concentration of the fatty acid chloride, affect the functional properties of the PVOH films. The effects of the chemical grafting have already been described and are not further discussed here (see Stinga, 2008; Schmid et al., 2012, 2014). Strategies for surface hydrophobization are described elsewhere (Müller et al., 2017). The aim of this work was to improve the solubility of grafted films.

## Materials and methods

### Sample preparation

The studies were performed on a single layer PVOH film (50 μm; ±5 μm) manufactured using a flat extrusion process and then subjected to chemical grafting. The PVOH pellets were produced by Plásticos Hidrosolubles S.L. (Rafelbuñol, Valencia, Spain) and supplied by SOKUFOL FOLIEN GmbH (Limburg, Germany). The properties of the PVOH, such as the degree of hydrolysis, were unknown. The chemical grafting was performed using the transfer method according to Stinga (2008).

#### Extrusion of the monolayer PVOH film

The PVOH film was manufactured by a flat extrusion process at the Fraunhofer IVV (Freising, Germany). The PVOH pellets (product description: B007) were supplied by Sokufol Folien GmbH (Limburg, Germany). The PVOH film had cold water soluble characteristics, resulting in dissolution when the film is immersed in cold water of about 20°C.

The extrusion of the cold, single layer PVOH film was carried out on a single screw extrusion unit (Type E30Px30L/D, Dr. COLLIN GmbH, Ebersberg, Germany). After the extrusion unit there was a chill-roll/calendar-system for further processing of the film with an edge trim and a rewind station (Collin GmbH, 2006; Wagner et al., 2014).

Due to the hygroscopic nature of PVOH (Olabisi, 1997) and the high temperature in the extruder, the pellets were dried (Wagner et al., 2014) in an oven at 40°C for 20 h prior to the extrusion process. The pellets must have a water content of less than 0.5% (based on experience) for extrusion of a polymer film without defects. After the extrusion process the PVOH film was stored at 23°C at a relative humidity of 50% to ensure the stability of the film.

#### Chemical grafting using a fatty acid chloride

Chemical grafting was used (Samain, 2002; Stinga, 2008; Schmid et al., 2012, 2014) to modify the polymer film surface in order to increase its water repellency. The single layer PVOH film was chemically grafted with palmitoyl chloride (C_16_).

**Figure 1:**
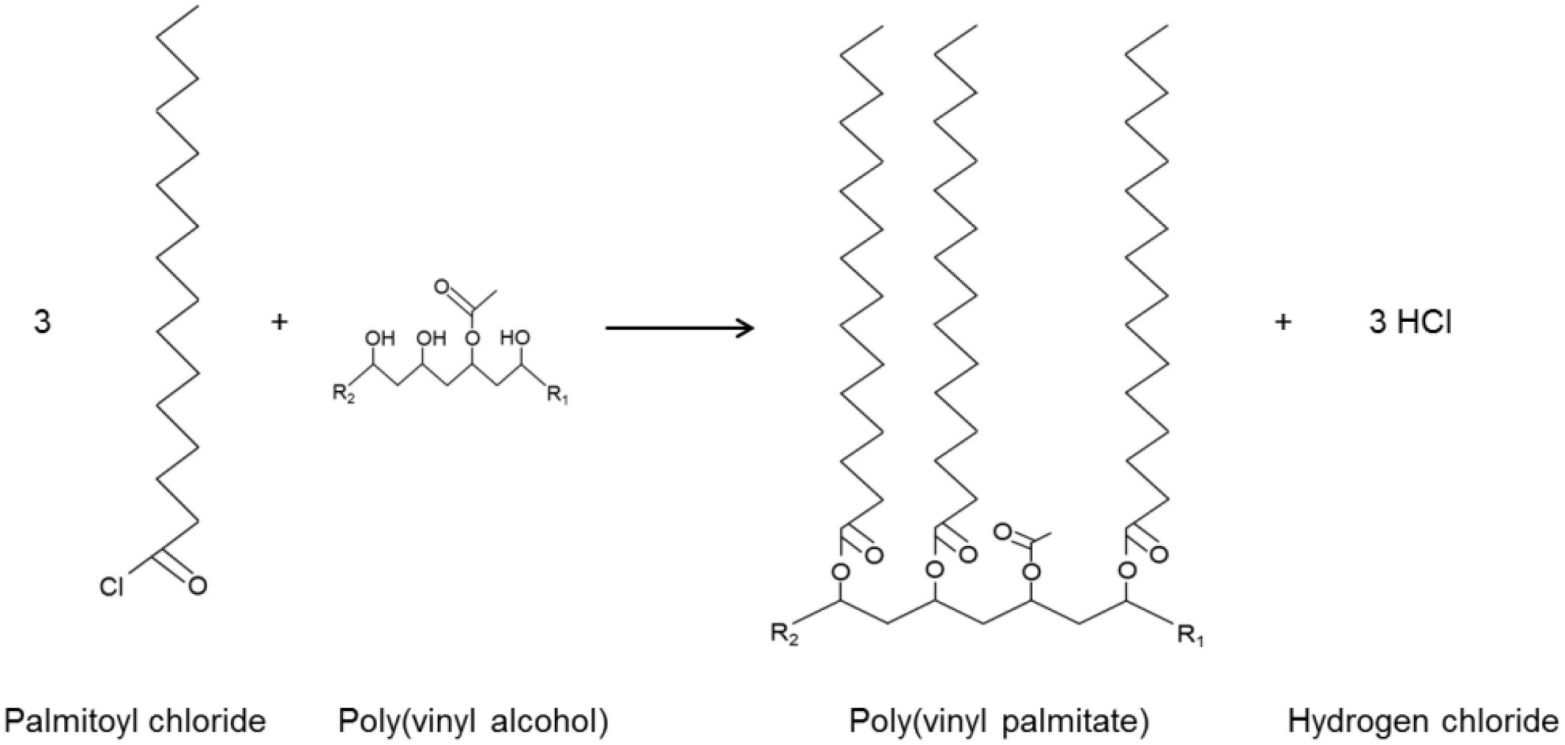
Esterification of PVOH involving reaction between palmitoyl chloride (fatty acid chloride) and the hydroxyl groups of the PVOH film (Schmid et al., 2012), resulting in polyvinyl palmitate and hydrogen chloride; R_1_ and R_2_ represent the continuation of the PVOH chain.

Figure 1 shows the esterification process. Hydrochloric acid is formed during the process and released into the air.

The chemical grafting was achieved using the transfer method according to Stinga (Stinga, 2008; Schmid et al., 2014). Table 1 shows the materials/equipment used for the grafting process.

**Table 1:** Materials/equipment used for the chemical grafting process.

**Process**	**Material/equipment**	**Manufacturer**
Application of the solution	Filter paper no. 2	Whatman GmbH, Dassel, Germany
Container for the solution	Glass dish	SCHOTT AG, Landshut, Germany
Drying	Oven	Heraeus Holding GmbH, Hanau, Germany
Substrate	PVOH film	Fraunhofer IVV, Freising, Germany
Weighing the chemicals	Microbalance	Sartorius AG, Göttingen, Germany
Time measurement	Stopwatch	Carl Roth GmbH + Co. KG, Karlsruhe, Germany
Solvent	Petroleum ether	Merck KGaA, Darmstadt, Germany
Fatty acid chloride	Palmitoyl chloride	Merck KGaA, Darmstadt, Germany

The grafting of the films was performed using differing process parameters. Polymer films with high hydrophobicity indicated a successful grafting process. The parameters that were varied were the concentration of the fatty acid chloride (1, 2, and 3%), the grafting temperature (90–150°C), and the grafting time (2–20 min).

A statistical experimental design was generated (Visual XSel 13.0, CRGRAPH GbR, Starnberg, Germany) as shown in Table 2.

**Table 2:** Experimental design.

**Sample number**	**Concentration % (w/w)**	**Temperature °C**	**Time min**
1	1	110	14
2	1	130	8
3	1	90	2
4	1	90	20
5	1	150	20
6	1	150	2
7	3	150	2
8	3	130	20
9	3	90	8
10	3	120	11
11	3	120	11
12	3	120	11
13	5	150	14
14	5	90	2
15	5	150	20
16	5	150	2
17	5	90	20
18	5	110	20
Ref.	0	23	0

*A D-optimal and cubic experimental design led to a reduced number of experiments that allowed the acquisition of significant information about the success of the grafting. To compare the results, a reference sample (not processed) was tested*.

The chemical grafting was performed using the following method according to Stinga (2008). A mixture of defined amounts of petroleum ether and fatty acid chloride (according to Table 2) were applied to a filter paper by immersing it in the solution. The PVOH film, of DIN A4 size, was placed on a metal plate. After drying the filter paper for a short time (about 30 s, 23°C, 50% r.h.), it was placed on top of the PVOH film and fixed with clips. This was then put in an oven at a defined temperature. In the oven the reagent diffused into the PVOH film and the grafting process took place. The surface of the PVOH adsorbed the fatty acid chloride, chemical reaction took place, and the petroleum ether diffused. A byproduct of this esterification process is hydrochloric acid (HCl) which is liberated into the gas flow. Therefore, the oven was placed in a deduction unit (Samain, 2002; Schmid et al., 2012, 2014; Stinga, 2008).

**Figure 2:**
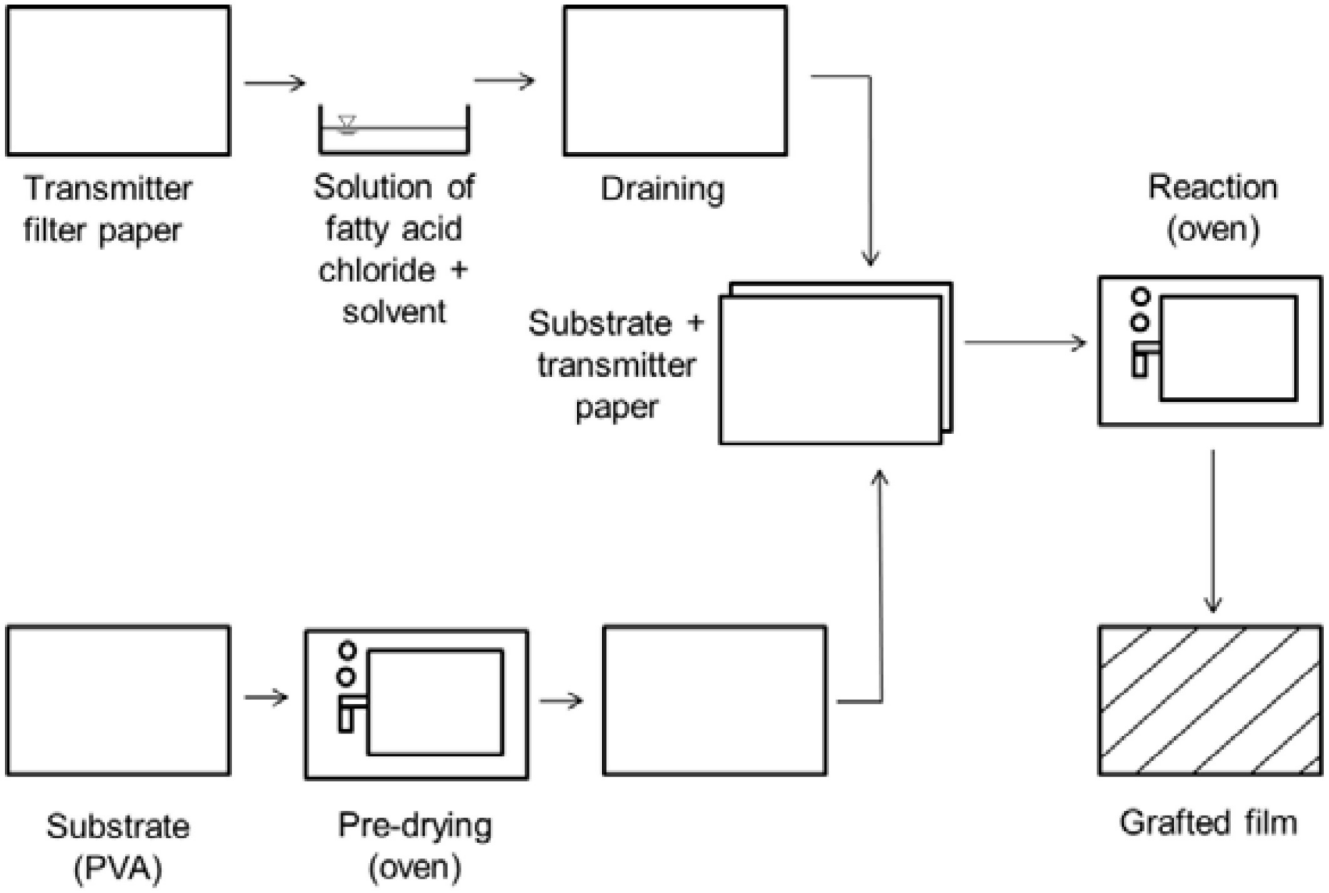
Schematic representation of the grafting process according to Schmid et al. (2012).

Figure 2 shows the technical grafting process on a laboratory scale according to Schmid et al. (2012).

Once the grafting process was finished, the substrate film had a nano-scale hydrophobic layer which was dependent on the esterification/grafting density, the concentration of the fatty acid chloride, and the grafting temperature and time (Samain, 2002; Schmid et al., 2012, 2014).

### Statistical methods

To analyze the measurement results, the mean and standard deviation were calculated (Ronniger, 2013). All the results were all checked for normal distribution according to Kolmogorov-Smirnov (α = 0.5) (Feldman and Valdez-Flores, 2010) or Anderson-Darling (α = 0.05) (Wilker, 2013), depending the number of test repeats per sample. The results were tested for outliers using the statistical outlier test according to Hampel (α = 0.05) (Rousseeuw and Leroy, 1987). To compare two or more values Student's *t*-test was used (*p*-value: 0.05) (Zöfel, 2001).

The water reactivity test and water vapor transmission results were statistically analyzed using Visual XSel 13.0 software (CRGRAPH GbR, Starnberg, Germany) (Ronniger, 2013). The analyses (d-optimal; regression analysis/ANOVA; Girden, 1992; cubic design with interactions) were performed on the samples outlined in Table 2.

### Sample characterization

#### Water reactivity test

The water reactivity test was performed on the grafted PVOH films and on an untreated sample as reference. The water reactivity test provides detailed information about the solubility of PVOH films, giving visible results within a short period of time. For this, 1 ml of distilled water was dropped onto the polymer film (cut into 12 × 12 cm samples). The film was then placed on a beaker (200 ml, d = 7.5 cm) and clamped with a rubber ring (article number: 10105419; d = 5 cm).

Both sides of the polymer film were monitored separately. The temperature of the water was 20°C. The time until the onset of visible reaction of the film material and the time for complete dissolution of the film were measured. Once the film dissolved the water dropped in the beaker and this defined the end time. The maximum test time was set at 30 min (1,800 s). The materials/equipment used for the water reactivity test are listed in Table 3.

**Table 3:** Materials/equipment used for the water reactivity test.

**Process**	**Material/equipment**	**Manufacturer**
Receptacle for distilled water	Beaker (200 ml)	SCHOTT AG, Landshut, Germany
Application of distilled water	Disposable pipette	Fisher Scientific GmbH, Schwerte, Germany
Time measurement	Stopwatch	Carl Roth GmbH + Co. KG, Karlsruhe, Germany
Temperature measurement	Thermometer	Brand GmbH + Co. KG, Wertheim, Germany
Receptacle for the film	Beaker (200 ml)	SCHOTT AG, Landshut, Germany
Clamping the film on the beaker	Rubber ring	Pelikan Vertriebsgesellschaft mbH & Co. KG, Hannover, Germany
Heating the distilled water	Heating plate	Franz Morat GmbH + Co. KG, Eisenbach, Germany

**Figure 3:**
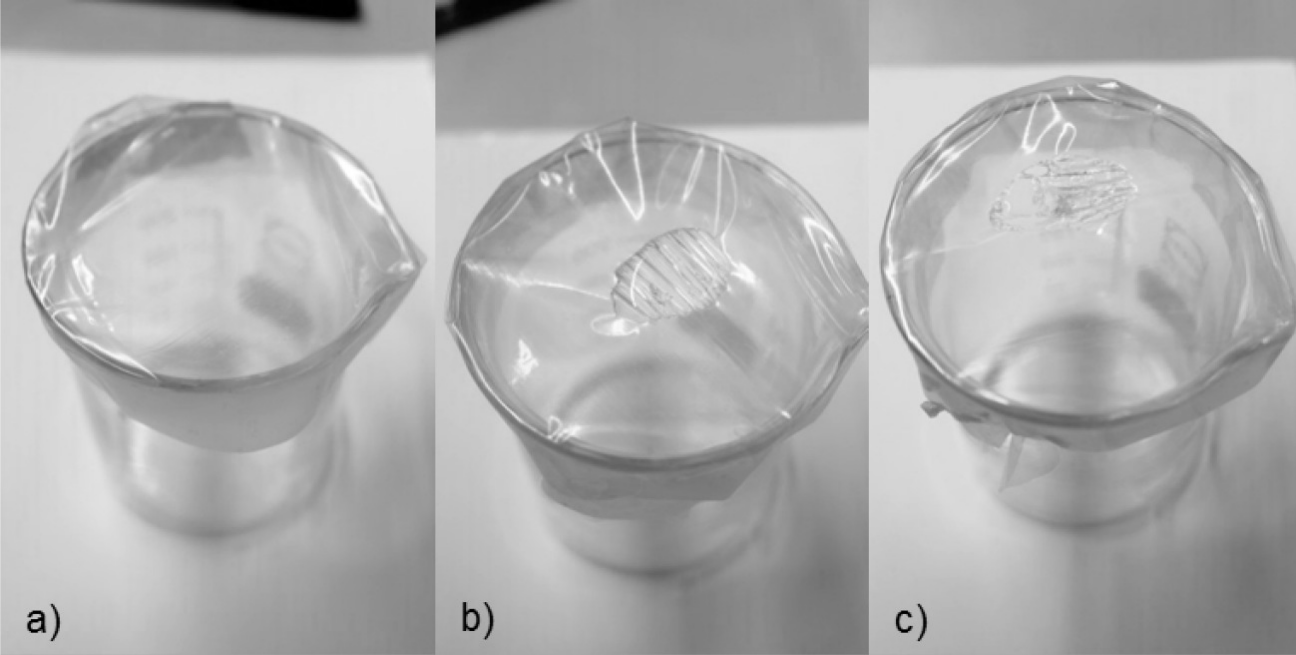
Stages of the water reactivity test (source: Fraunhofer IVV). **(a)** shows the PVOH film over the beaker, fixed with a rubber ring. When 1 ml was placed on top of the film, the film started to react as shown in **(b)**. This time was defined as “water reactivity time 1” (WRT1). Depending on the water soluble properties, the film eventually breaks and the water drips into the beaker. This was defined as “water reactivity time 2” (WRT2). A film with the leak is shown in **(c)**. It was found that the times for reaction and break of the film varied significantly depending on the grafting process, polymer composition, and the thickness of the film.

To bring the distilled water up to the desired temperature the beaker of water was heated on a heating plate. The temperature of the distilled water was measured using a thermometer. Figure 3 shows the various reaction steps during the water reactivity test.

#### Solubility test

The solubility test was performed on the grafted PVOH films to determine their ability to dissolve in water. The time until the film broke and the time until the film completely dissolved (no visible residues) were measured. Visual monitoring was used to determine when the film broke and had dissolved. The solubility test considers the whole film (both sides) in a single test. Table 4 shows the materials/equipment used for the solubility test.

**Table 4:** Materials/equipment used for the solubility test.

**Process**	**Material/equipment**	**Manufacturer**
Container for distilled water	Beaker (1 l)	SCHOTT AG, Landshut, Germany
Immersion of the film in water	Slide frame	Hama Hamaphot Hanke & Thomas GmbH & Co. KG, Monheim, Germany
Time measurement	Stopwatch	Carl Roth GmbH & Co. KG, Karlsruhe, Germany
Temperature measurement	Thermometer	Brand GmbH & Co. KG, Wertheim, Germany
Stirring the water	Magnetic stirrer	Franz Morat & Co. GmbH Elektro-Feinmechanik & Maschinenbau, Eisenbach, Germany
Heating the distilled water	Heating plate	Franz Morat & Co. GmbH Elektro-Feinmechanik & Maschinenbau, Eisenbach, Germany

The film samples were cut into squares (5 × 5 cm) and clamped into the frame. This resulted in a defined film surface of 24 × 36 mm. Each film was tested five times and the times for disintegration and total dissolution were measured. The beaker was filled with ~900 ml distilled water and placed on a magnetic stirrer (450 rpm). The temperature of the water was 20°C (±2°C), depending on the cold water solubility characteristics of the PVOH film. The frame with the clamped film was put into the stirred water and completely immersed. This moment was defined as the start of the time measurement.

Film disintegration was defined as a break or tear in the film, independent of the size of the defect (visual monitoring). A small break/tear was sufficient to declare the film as disintegrated. Dissolution here means complete dissolution of the polymer film. The dissolution time is defined as the time required for the film in the frame and the small particles in the water to completely dissolve. The maximum test time was set at 30 min (1,800 s).

#### Contact angle measurement

The contact angle of a liquid characterizes the surface with regard to its hydrophobicity, surface tension, and surface energy (Nalwa, 2002).

The contact angle is determined by placing a drop of a liquid on the surface and then taking an image and measuring the angle. From the results the surface energy can be calculated and statements can be made about the hydrophobicity of the surface.

The following equation describes the correlation between the interfacial tension and the contact angle of the liquid.

$$\begin{array}{l} \gamma \left(lv\right)\;x\;cos\theta \left(y\right)=\gamma \left(sv\right)-\gamma \left(sl\right) \end{array}$$

**Table:** 

γ (lv)	Liquid-vapor interfacial tension	[kg/s^2^]
γ (sv)	Solid-vapor interfacial tension	[kg/s^2^]
γ (sl)	Solid-liquid interfacial tension	[kg/s^2^]
θ (y)	Contact angle	[°]

The surface energy is the sum of polar and disperse forces. Materials with hydrophilic properties have high surface energy, leading to a low contact angle and good wetting of the film. Hydrophilic materials also have a higher fraction of polar forces than disperse forces (Schmidt, 1999).

In this study, the surface energy was measured with a Drop Shape Analyzer DSA100 manufactured by KRÜSS GmbH, Hamburg, Germany. DAS I-Software was used and the test liquids were water (H_2_O; SE = 72.8 mN/m), diiodomethane (CH_2_I_2_; SE = 51.6 mN/m), and ethylene glycol (C_2_H_6_O_2_; SE = 47.7 mN/m). For the test, 3 μl of the liquid was dropped onto the film surface at a dosing speed of 20 μl/min. The method was static, namely the contact area between the liquid and film did not change during the measurement. The evaluation was performed with the method of Owens-Wendt-Rabel & Kälble (Brockmann, 2005; Sudarshan and Stiglich, 2005; Günter and Jakob, 2015). To ascertain the free surface energy of the solid, at least two liquids with known disperse and polar fractions of the surface tension were required. It had to be ensured that one liquid had a polar fraction greater than zero (Kaelble, 1970).

Due to the water-soluble characteristics of PVOH, the contact angle could not be measured at equilibrium. The moment the drop was placed on the surface, the image was taken and the contact angle was measured.

#### Water vapor transmission rate measurement

Products which are vulnerable to moisture must be wrapped in secure packaging to protect them from humidity (Kadoya, 1990). This is achieved by using a high barrier film to increase the shelf life of the product (Ebnesajjad, 2013b). The polymer used for the packaging film (here PVOH) is important here. The polymers can have differing orientation and different degrees of crystallinity (Jenkins and Harrington, 1992).

The general method used to determine the water vapor transmission rate is the gravimetric method according to DIN 53122-1 (D. I. f. N. e.V., 2001).

Four round samples of the same size are cut out of the film and are masked on both sides. These are placed on aluminum cups which are lubricated to improve the seal. These cups are filled with a water vapor absorber such as silica gel. The lids of the cups are then fixed by six screws. To improve the stability, the lids have sealing rings. The starting weight is measured using a balance. The cups are then placed in a climate chamber set at room temperature and humidity. The moisture in the air permeates through the unmasked area of the sample to the silica gel, which absorbs the water and increases in weight (Brown, 1999; D. I. f. N. e.V., 2001; Mehyar and Han, 2004; McKeen, 2006).

The water vapor transmission rate was calculated using the following formula:

$$\begin{array}{l} WVP=\;\frac{WVTR}{\Delta p\left(H_{2}O\right)}*d\;\left[\text{g}\mu \text{m}{\text{m}}^{-2}{\text{d}}^{-1}\text{ba}{\text{r}}^{-1}\right] \end{array}$$

**Table:** 

WVP	Water vapor permeation coefficient	[g μm m^−2^ d^−1^ bar^−1^]
WVTR	Water vapor transmission rate	[g m^−2^ d^−1^]
Δp (H_2_O)	Partial pressure difference of water vapor between the films	[bar]
d	Thickness of the film	[μm]

The samples were weighed four times in 48 h. After a certain time the weight becomes constant which denotes the end of the measurement (Brown, 1999). In this study the test conditions were 23°C and a relative humidity ranging from 50 to 0%.

## Results

### Water reactivity

The water reactivity test was performed on the grafted and non-grafted side of the polymer film. In this study, the water reactivity time t2 was determined (time until the film breaks). If no reaction was observed after 30 min (1,800 s), the test was stopped.

**Figure 4:**
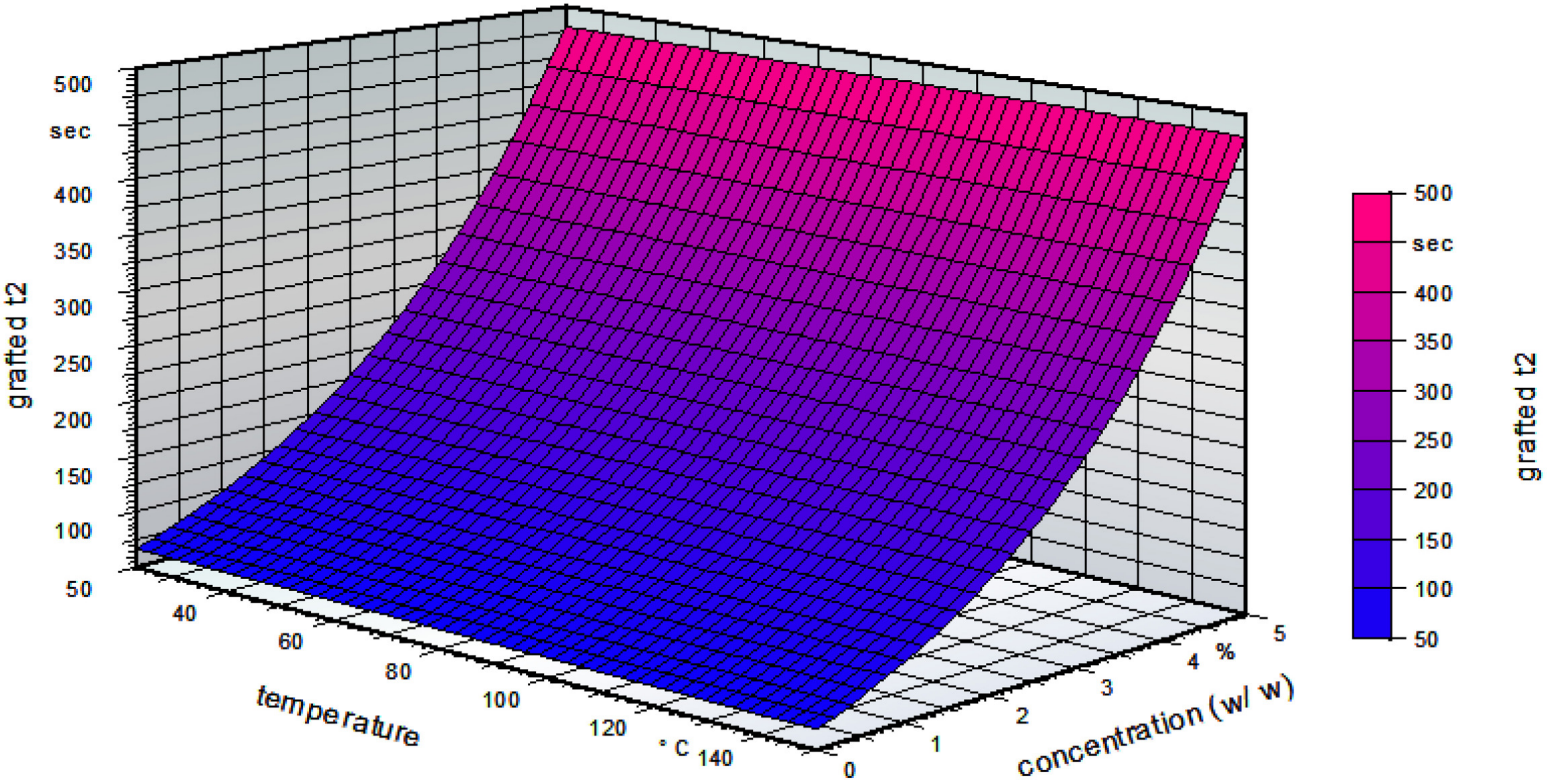
Water reactivity test on the grafted side of the film for a standardized grafting time of 10 min (water reactivity time t2, grafted t2; w/w, weight/weight).

Figure 4 shows the influence of the temperature and the concentration for a standardized time of 10 min on the grafted side of the film. It can be clearly seen that the temperature did not influence the grafting process. With increasing concentration, the water reactivity time increased.

**Figure 5:**
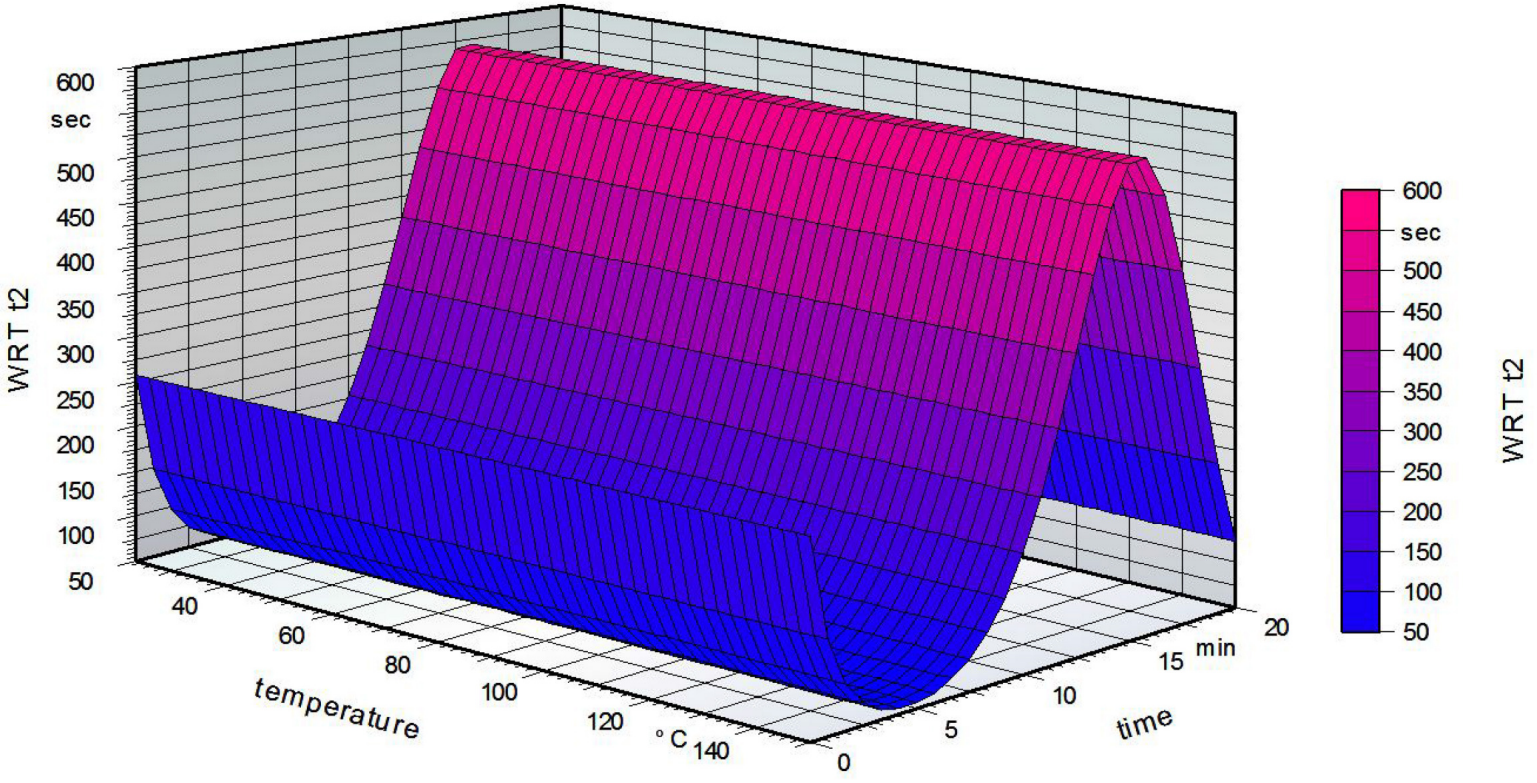
Water reactivity test on the grafted side of the film for a standardized fatty acid concentration of 2.5% (w/w, weight/weight).

Figure 5 strengthens the assumption that increasing temperature does not influence the hydrophobicity of the film. Increasing the time led to a small decline at first, but then a marked increase. The maximum was reached for a grafting time of 15 min, followed by a decrease at 20 min. It is possible that for low grafting times the fatty acid can only partially attach to the PVOH surface. Short heat treatment of the PVOH could then lead to a structure with decreased stability. In general, heat treatment of PVOH leads to a chemical change in the crystallinity of the polymer (Sakurada, 1985). Another possibility is that remaining water inside the PVOH film could decrease the solubility but is liberated on longer grafting times in the oven. The crystallinity of the PVOH is possibly raised by thermal treatment, resulting in a more insoluble film (Olabisi, 1997).

**Figure 6:**
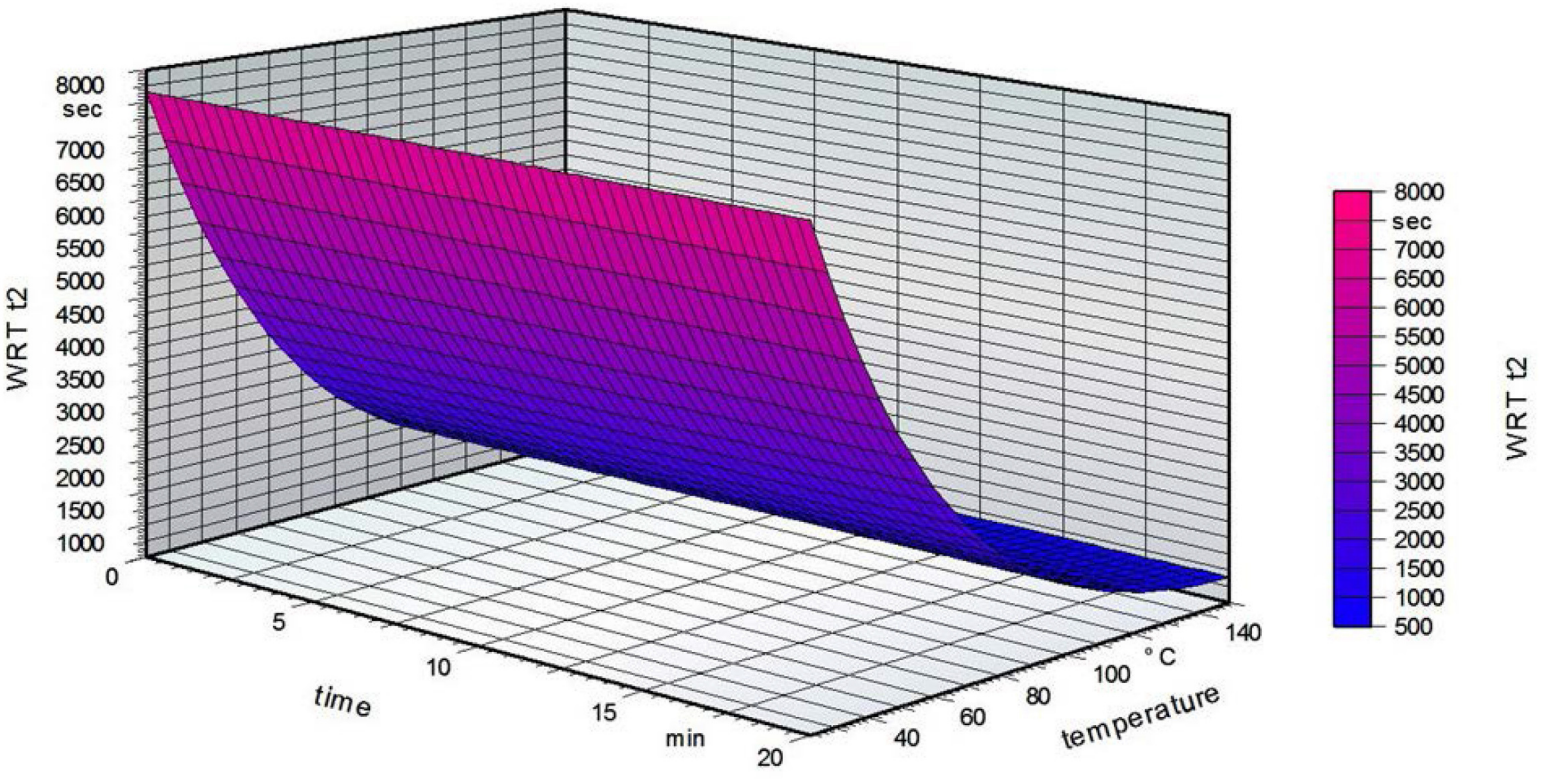
Water reactivity test on the non-grafted side of the film for a standardized fatty acid concentration of 2.5%.

Figure 6 shows that the grafting time did not influence the water reactivity time t2 on the non-grafted PVOH film side for a standardized fatty acid concentration of 2.5%. The temperature has a marked influence, with a decreasing water reactivity time at higher temperatures. These results are opposite to what was expected, namely that a high grafting temperature leads to a higher water reactivity time t2 (Samain, 2002). The following table summarizes the results of the water reactivity test.

According to Table 5, it is assumed that the concentration of the fatty acid chloride had the biggest influence on the film hydrophobicity, both on the chemically modified and non-modified film side. The time in the oven only influenced the water repellency on the grafted side. A possible reason for this could be that the grafting reaction took place on the grafted side and not on the other side of the film using the transfer method (Schmid et al., 2012). The temperature influenced the water repellency of the non-grafted side (and probably the grafted side) (Byron and Dalby, 1987).

**Table 5:** Influence of the three parameters on WRT t2 on the grafted and non-grafted sides of the film; x, significant influence; o, minor influence; –, negative effect; +, positive effect.

**Water reactivity test**	**Influence of**		
**(t2)**	**Temperature**	**Time**	**Concentration**
Grafted side	o	x/+	x/+
Non-grafted side	x/–	o	x/+

### Solubility

For analysis of the solubility test results, two of the three process parameters were kept constant. This allowed evaluation of the influence of one single process parameter. The solubility time 2 (sol t2) was used for the analysis because it was more objective than solubility time 1 (sol t1). The means were calculated and marked with the relevant standard deviations. The differences in the solubility time means were statistically analyzed where possible using Student's *t*-test. Sample values having no standard deviation and variance could not be analyzed using statistical methods (not marked with letters). Different letters indicate a significant difference between the measured values. To denote significant differences, the solubility time 2 (sol t2) was marked with capital letters and the solubility time 1 (sol t1) with small letters.

**Figure 7:**
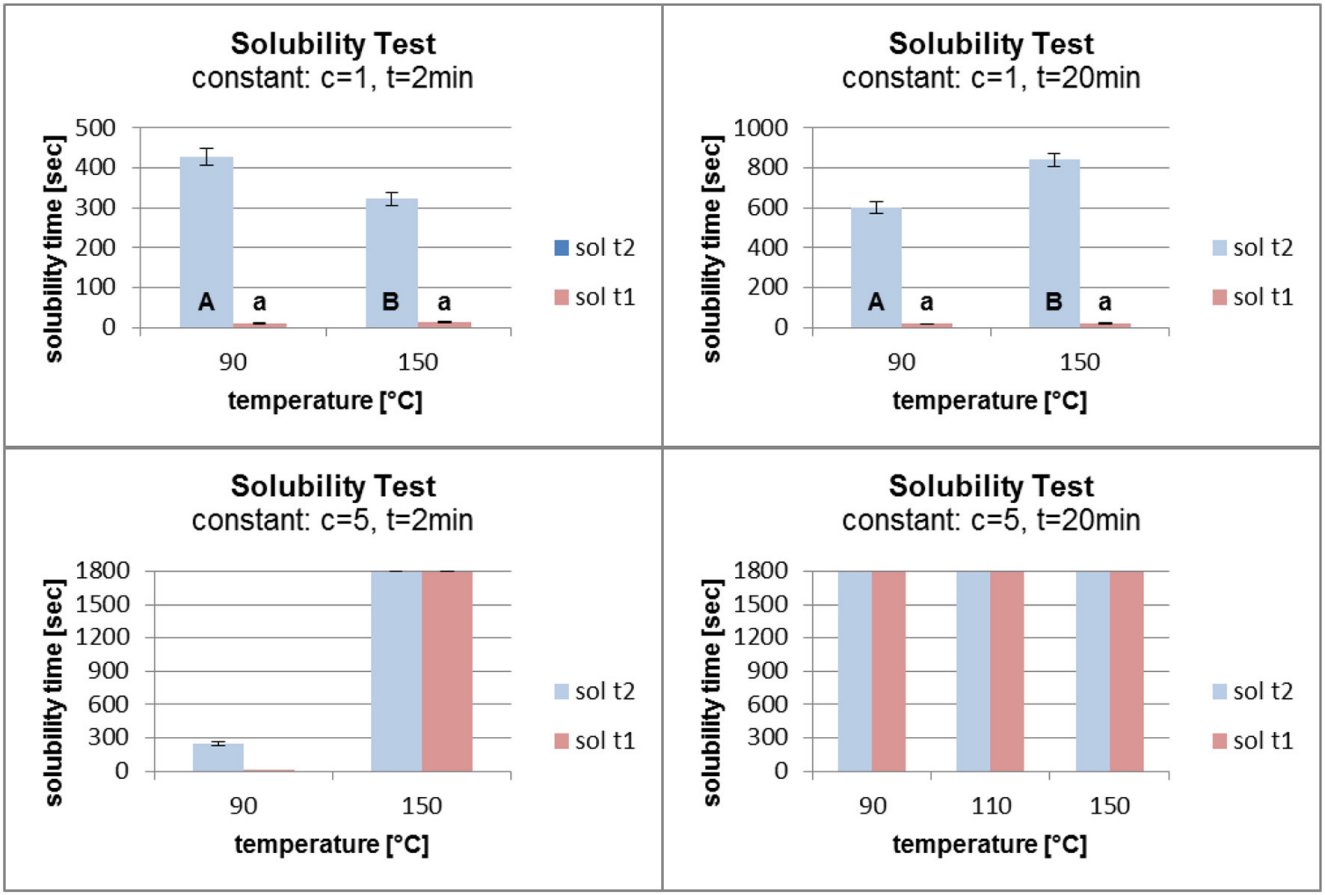
Solubility at constant fatty acid concentration (c) and time (t) (sol t1, time for disintegration; sol t2, time for dissolution).

Figure 7 shows that an increase in temperature from 90°C to 150°C had no major influence on the solubility of the grafted polymer film for a fatty acid concentration of 1%. When the time in the oven was raised from 2 to 20 min, a small increase in the solubility time with increasing temperature was observed. For a concentration of 5% and 2 min in the oven, increasing the temperature had a major influence on the polymer film. The solubility time of the film increased from about 200 s to the maximum of 1,800 s. When the time in the oven was set at 20 min, the temperature had no influence on the film stability. The solubility time reached 1,800 s without being dependent on the oven temperature.

**Figure 8:**
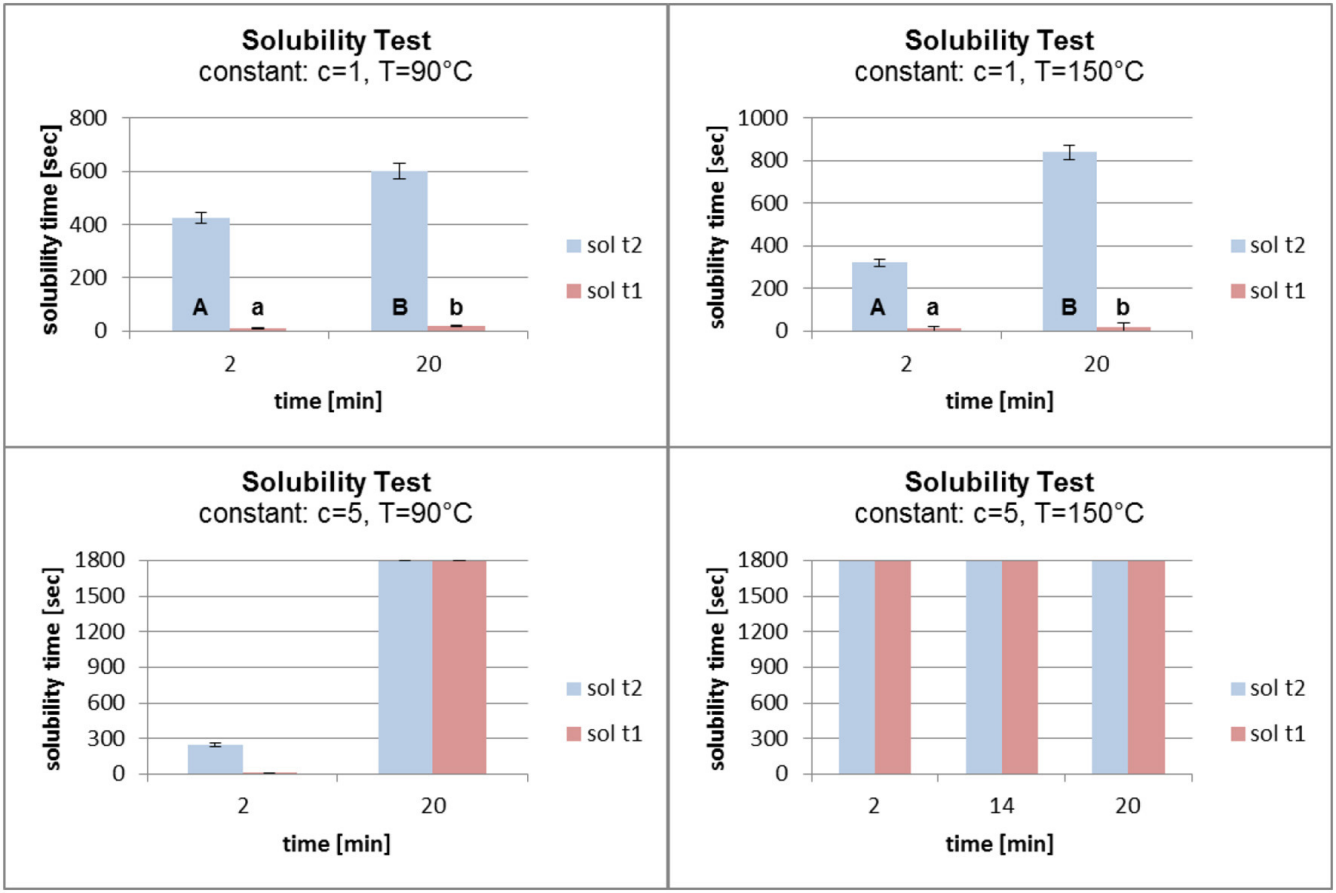
Solubility at constant fatty acid concentration (c) and temperature (t) (sol t1, time for disintegration; sol t2, time for dissolution).

In Figure 8 the concentration and the temperature were kept constant. An increase in the grafting time from 2 to 20 min (at a concentration of 1% and a temperature of 90°C) led to a small increase in the solubility time. A more significant effect was seen at a constant temperature of 150°C. The solubility time increased from about 300 s to over 800 s. Increasing the grafting time (at a constant concentration of 5% and a temperature of 90°C) had an enormous effect on the solubility time which increased from about 250 s to the maximum of 1,800 s. When the temperature was kept constant at 150°C, the different grafting times did not influence the solubility time. In each case it reached the maximum of 1,800 s.

**Figure 9:**
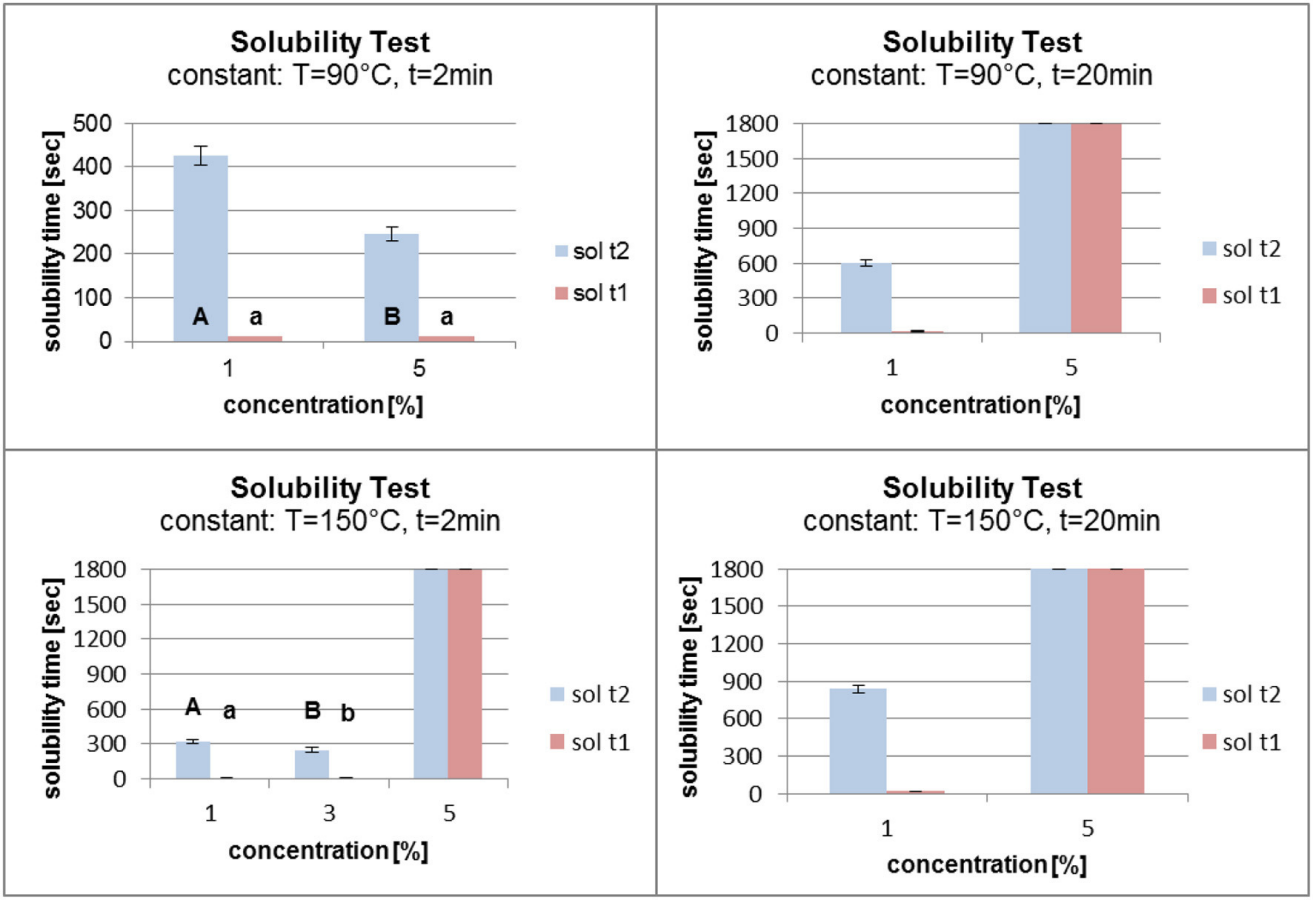
Solubility at constant temperature (T) and time (t) (sol t1, time for disintegration; sol t2, time for dissolution).

Figure 9 shows the results of the solubility test at constant grafting temperatures and times. A temperature of 90°C and a time of 2 min at different grafting concentrations led to a reverse grafting effect, which was not expected. The solubility time of the film decreased, which can be explained by the need for higher energy input for the higher concentration of fatty acid chlorides than for the lower concentration in order to attach the molecules to the surface. It is conceivable that at a lower concentration and with the same energy input the fatty acid chloride attached faster to the surface and reacted better with the PVOH film. When the grafting time was raised to 20 min, raising the concentration from 1 to 5% affected the solubility time which increased from about 550 to 1,800 s. At high temperature (150°C) and short grafting time (2 min) the concentrations of 1 and 3% gave almost the same solubility results. On increasing the concentration from 3 to 5% the solubility time increased markedly from 300 s to the maximum of 1,800 s. When the grafting time was kept constant at 20 min, an increase in the concentration led to a better grafting effect. The solubility time increased from 800 s to the maximum.

In conclusion it can be stated that the duration of the grafting process in the oven had the largest influence on the film solubility. For a grafting time of 20 min, the concentration of the fatty acid chloride and the temperature had little effect the hydrophobicity. Concentrations of 1 and 3% fatty acid chloride did not lead to a polymer film which reached the maximum time in the solubility test. When the polymer film was kept in the oven for 2 min, reaction probably did not occur except at a temperature of 150°C. A fatty acid chloride concentration of 5% was also not able to compensate the short grafting time. One side of the film was chemically modified, the other side was not. According to Byron et al., an increase in stability due to the thermal treatment influences the solubility (Byron and Dalby, 1987). This effect was also observed in these studies.

### Surface energy by contact angle measurement

**Figure 10:**
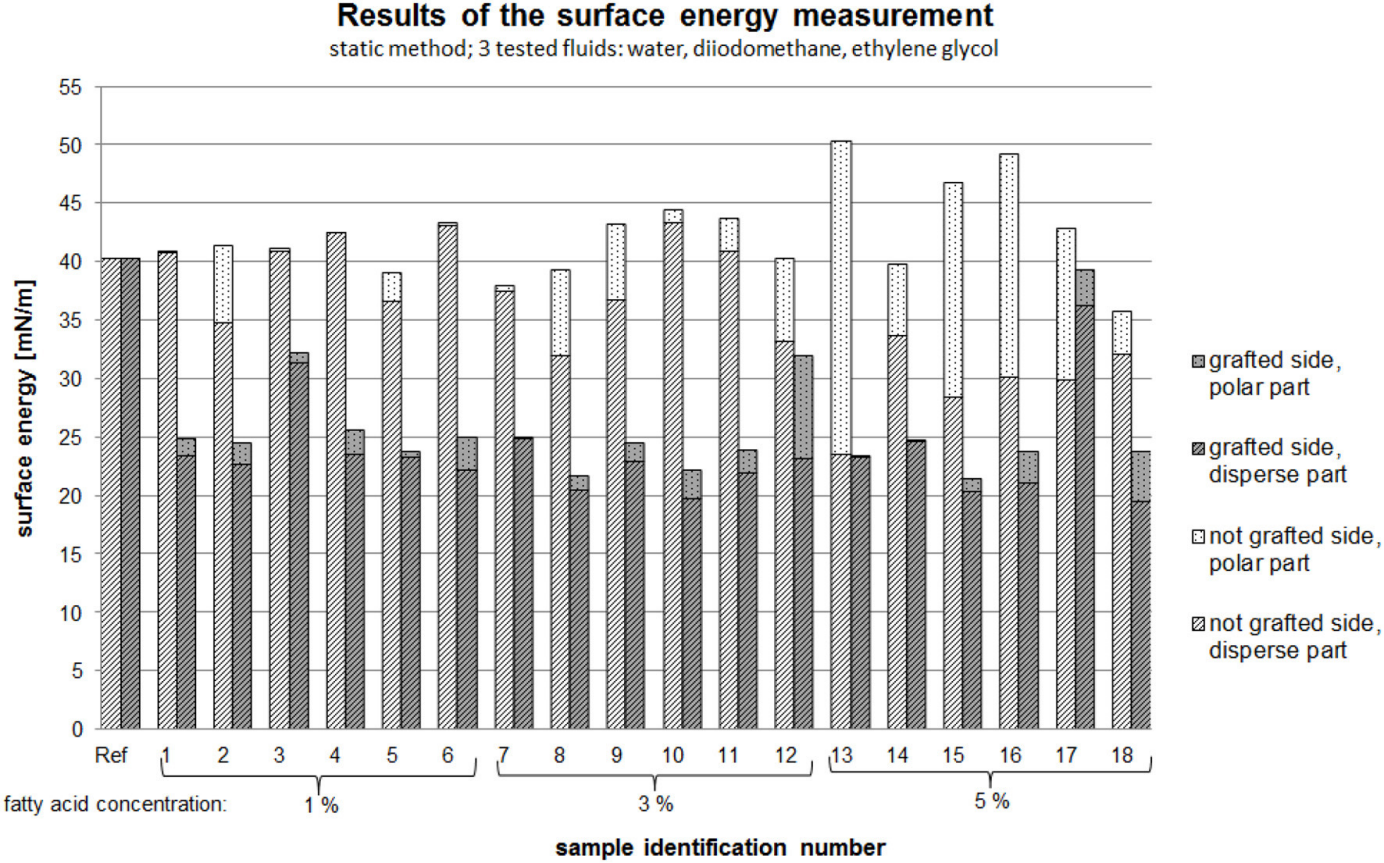
Surface energy as a function of the polar and disperse forces and the different concentrations of the fatty acid chloride.

The effect of the process parameters on the surface energy was analyzed, with consideration given to both the grafted side and non-grafted side of the PVOH films. Figure 10 shows the surface energy as a function of the polar and disperse forces and the different concentrations of the fatty acid chloride.

Independent to the process parameters, it was observed that the non-grafted side of the film had higher polar forces than the grafted side of the film (see Figure 10). Based on theoretical knowledge, hydrophilic materials have higher polar forces than disperse forces and this was evident in the results of this work. However, a higher polar fraction of the surface energy on the non-grafted side of the film was not observed in every sample.

According to Schmid et al. (2012), grafting of the PVOH film leads to a hydrophobic surface on the grafted side of the film and this was confirmed by our results. With increasing fatty acid concentration the surface energy increased, resulting in a more hydrophobic surface. With increasing concentration, the polar fraction of the surface energy on the non-grafted side increased. The surface energy of the grafted side had a lower polar fraction than the non-grafted side.

The research work of in particular Byron and Dalby (1987) shows that controlled thermal treatment of PVOH films using defined process parameters leads to polymer films with defined solubilities. It was found that thermal treatment of PVOH films leads to higher crystallinity and improved film stability (Byron and Dalby, 1987). This can lead to films with lower surface energy.

An interesting observation was the result for sample 13 (process parameters: 5%, 150°C, 14 min). For this sample, the surface energy on the grafted side only consisted of a disperse fraction. Overall the non-grafted sides of the modified PVOH films were more hydrophilic than the grafted sides, which was desired.

### Water vapor transmission rate

The water vapor transmission rate of the modified films was measured to determine the improvement in the barrier properties. The hygroscopic nature of PVOH led to swelling of the film when the relative humidity was increased (Hodge et al., 1996). The surface modified by chemical grafting should have a lower water permeability due to the hydrophobic nature of the attached fatty acid groups.

**Figure 11:**
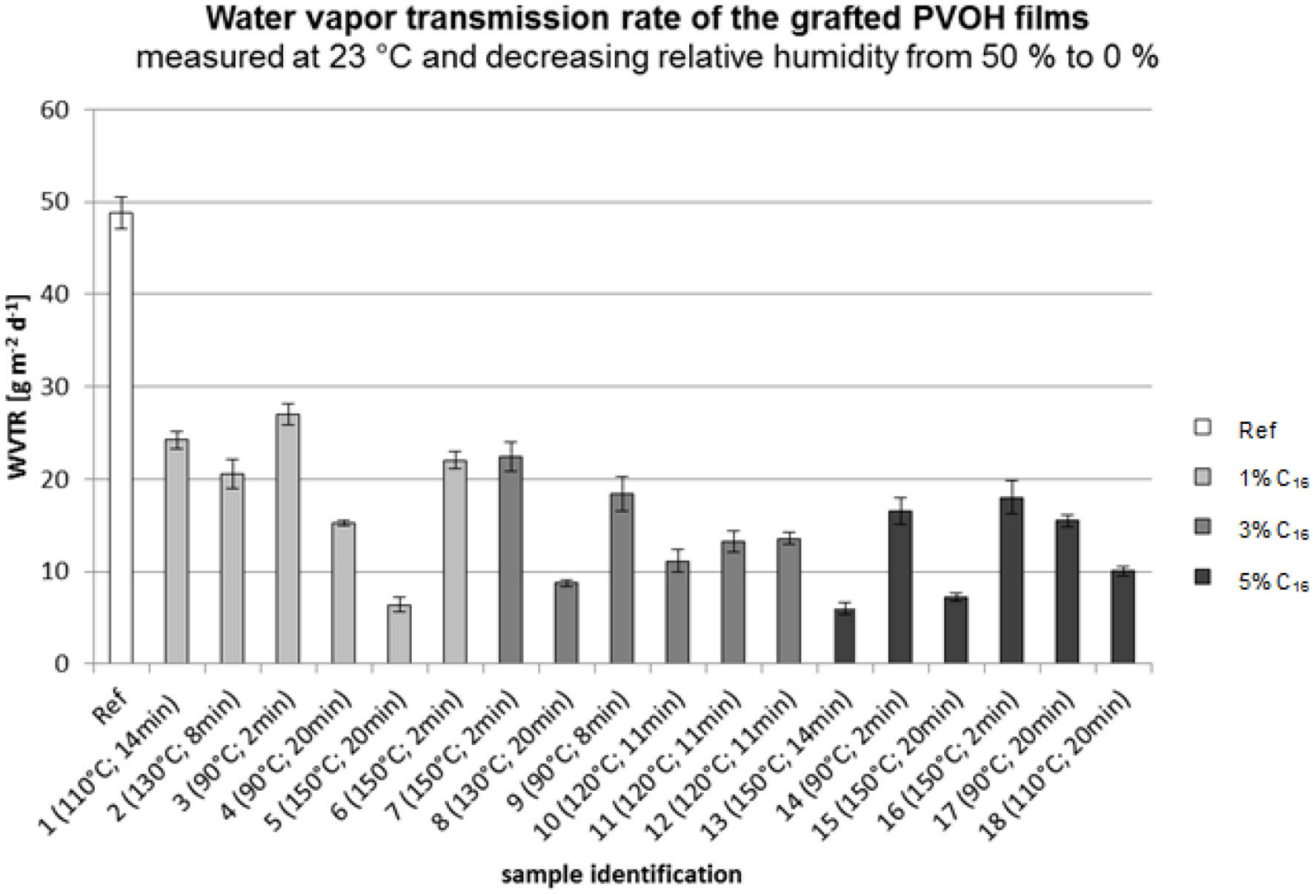
Water vapor transmission rate (WVTR) of the grafted PVOH films.

Figure 11 shows the water vapor transmission rate of the grafted PVOH films. The different fatty acid concentrations are marked in different shadings.

According to Figure 11, the reference sample (not modified) had the highest water vapor transmission rate. In general, the WVTR decreases only slightly with a higher fatty acid concentration. With regard to the grafting temperatures and times, the best barrier properties were obtained for sample 5 (1%; 150°C; 20 min), sample 8 (3%; 130°C; 20 min), sample 13 (5%; 150°C; 14 min), and sample 15 (5%; 150°C; 20 min). Based on this information, it can be stated that a high grafting time (>14 min) and high temperature (>130°C) lead to a low water vapor transmission rate. The effect of the fatty acid chloride concentration can be neglected because of its small influence compared to the effect of the grafting time and temperature.

**Figure 12:**
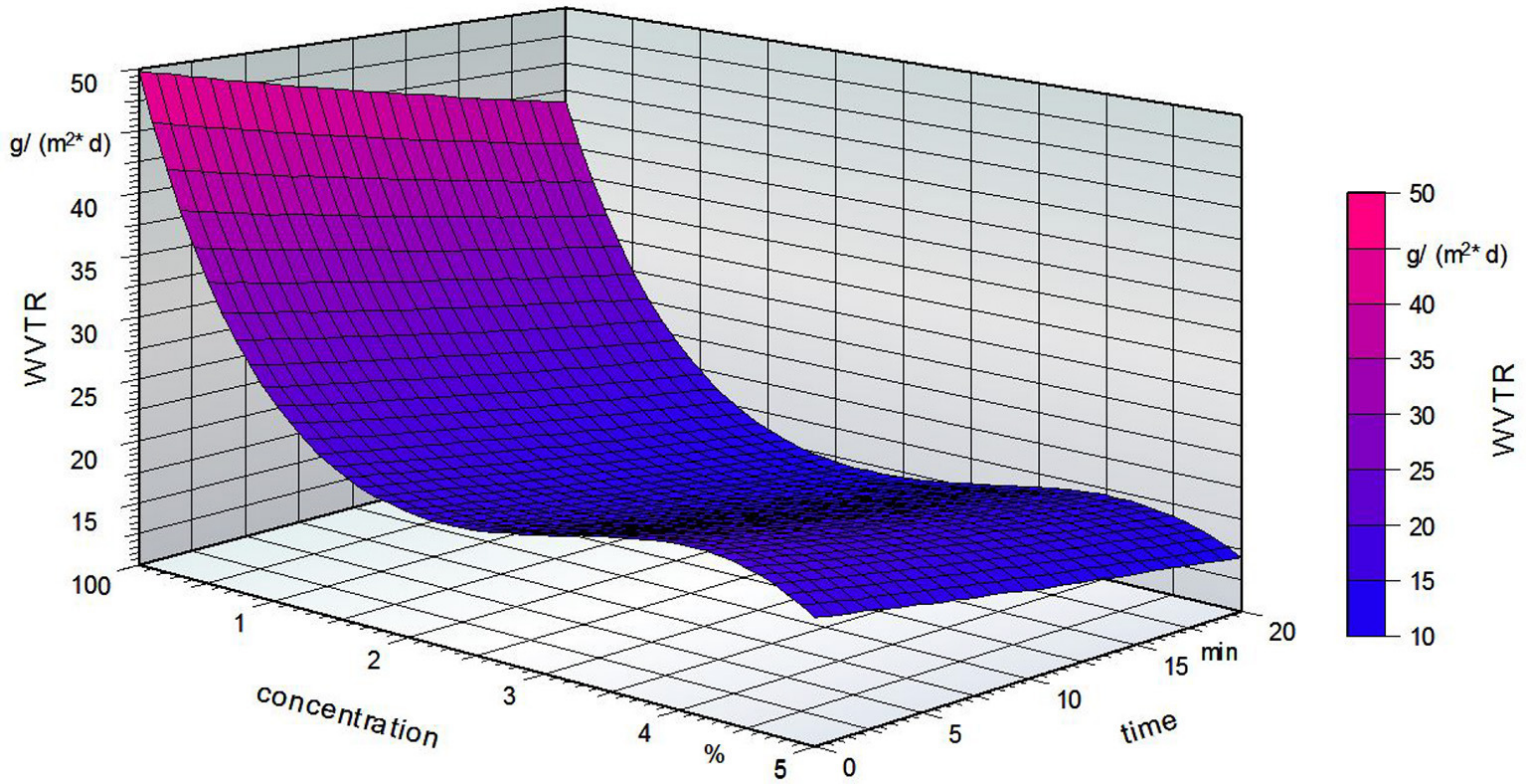
Water vapor transmission rate (WVTR) of the grafted PVOH films; standardized temperature of 86.5°C.

Figure 12 shows the influence of the fatty acid concentration and the grafting time on the water vapor transmission rate. An increase in the grafting time led to a lower WVTR. Compared to the reference sample, the presence of fatty acid groups led to a decrease in the WVTR. The fatty acid concentration had no effect on the barrier properties. It is seen that the WTVR decreases at a concentration of 2% followed by an increase. However, these changes are small compared to the influence of the time and temperature.

According to Beswick et al., PVOH films have excellent gas barrier properties and these properties diminish with increasing humidity (Beswick and Dunn, 2002). It is assumed that the thermal treatment of the PVOH film increases the stability of the film. It is possible that the molecules in the PVOH film become tempered which strengthens the film and improves the barrier properties. A high grafting temperature leads to an increase in the film crystallinity (Olabisi, 1997) which could increase the water vapor barrier of the PVOH film. According to Ellis (2008), heat treatment of PVA (polyvinyl acetate) leads to lower water permeability.

## Conclusions and outlook

The aim of the study was to develop a PVOH film with controlled water solubility by chemical grafting the film with palmitoyl chloride. The films were manufactured by flat film extrusion. To characterize the polymer films, barrier tests were performed.

The chemical grafting of the PVOH monolayer film led to a hydrophobic surface due to the chemically bound fatty acid. By using the transfer method it was possible to modify the film on one side while the other side was not processed and so made the film soluble. To identify the process parameters giving the best grafting effect on the film (most hydrophobic film), an experimental design was applied. This led to 18 different sets of parameters. To determine the effect of the process parameters, the grafted films were subjected to a solubility test, water reactivity test, water vapor transmission rate measurement, and contact angle measurement. The most suitable parameters for the grafting process were thus identified. Alteration of the process parameters allows adjustment of the film properties.

Table 6 shows a summary of the results. It can be seen that the concentration of the fatty acid chloride must be at least 3% to achieve good results. The grafting time in the must be at least 14 min. The temperature of the oven must be at least 130°C. The test methods showed that the best effect was achieved using different process parameters. Using this information the polymer film can be chemically grafted in order to influence and adjust its solubility.

**Table 6:** Process parameters giving the most hydrophobic surface for chemical grafting of a PVOH film with C16 fatty acid chloride.

**Main experiment**	**Desired results**	**Process parameters**		
		**Conc (%)**.	**Time**	**Temp**.
Water reactivity test	↑ Time	5	20 min	150°C
Solubility test	↑ Time	≥3	Low infl.	Low infl.
Surface energy	↓ Surface energy	5	20 min	150°C
WVTR	↓ WVTR	≥1	≥14 min	≥130°C

Furthermore, the film must be able to be welded. The grafting could influence the ability of the film to be welded to form a package and this aspect requires further investigation.

## Author contributions

MS, AH, and DS contributed edited the manuscript, conducted experiments, and wrote the paper. UG contributed by scientific discussions as well as interpretation of the results and revised the manuscript.

### Conflict of interest statement

The authors declare that the research was conducted in the absence of any commercial or financial relationships that could be construed as a potential conflict of interest.
